# Salud reproductiva: intersecciones entre tecnología y reproducción

**DOI:** 10.18294/sc.2025.5953

**Published:** 2025-10-03

**Authors:** Ana Lucía Olmos Álvarez, María Cecilia Johnson, Naara Luna, Rosa Martínez-Cuadros

**Affiliations:** 1 Doctora en Antropología Social. Investigadora asistente, Consejo Nacional de Investigaciones Científicas y Técnicas, con sede en la Universidad Nacional de Avellaneda, Buenos Aires, Argentina. analuciaolmos@gmail.com Consejo Nacional de Investigaciones Científicas y Técnicas Consejo Nacional de Investigaciones Científicas y Técnicas Universidad Nacional de Avellaneda Buenos Aires Argentina analuciaolmos@gmail.com; 2 Doctora en Estudios de Género. Investigadora asistente, Consejo Nacional de Investigaciones Científicas y Técnicas, con sede en el Centro de Investigaciones y Estudios sobre Cultura y Sociedad, Universidad Nacional de Córdoba, Córdoba, Argentina. cecilia.johnson@unc.edu.ar Consejo Nacional de Investigaciones Científicas y Técnicas Consejo Nacional de Investigaciones Científicas y Técnicas Centro de Investigaciones y Estudios sobre Cultura y Sociedad Universidad Nacional de Córdoba Córdoba Argentina cecilia.johnson@unc.edu.ar; 3 Doctora en Antropología. Profesora asociada, Universidade Federal Rural do Rio de Janeiro, Brasil. naaraluna2015@gmail.com Universidade Federal Rural do Rio de Janeiro Universidade Federal Rural do Rio de Janeiro Brazil naaraluna2015@gmail.com; 4 Doctora en Sociología. Investigadora postdoctoral, grupo GENI, Universitat de Barcelona, Barcelona, España. rosamartinezcuadros@gmail.com Universitat de Barcelona Universitat de Barcelona Barcelona Spain rosamartinezcuadros@gmail.com

## Introducción

En un siglo marcado por la vertiginosa innovación tecnológica y profundos cambios sociales, la salud reproductiva emerge como uno de los campos más dinámicos y disputados, reconfigurando no solo la intimidad individual, sino también las estructuras familiares, los derechos ciudadanos y las políticas estatales a escala global. La conjunción entre desarrollos científicos y mayores grados de acceso para el conjunto de la población a ciertas prácticas abrió horizontes insospechados en torno a la sexualidad, la reproducción y los proyectos parentales. Sin embargo, lejos de constituir un proceso homogéneo y universal, estos cambios se encuentran atravesados por regímenes normativos, historias nacionales, matrices religiosas y morales, y dispositivos regulatorios que configuran marcos diferenciales de posibilidad. En este sentido, y retomando la propuesta de Sarah Franklin y Marcia Inhorn^1^, podemos hablar de historias repronacionales: entramados específicos en los que tecnologías, cuerpos y políticas adquieren sentidos particulares y a menudo conflictivos.

Las tecnologías reproductivas han hecho posible la emergencia de experiencias y formas de agencia que, durante mucho tiempo, permanecieron invisibilizadas. A la vez, han planteado preguntas ineludibles acerca de los múltiples sentidos y formas de reconocimiento de los vínculos de parentesco^2^^,^^3^, los dilemas éticos y sociales en torno a los límites de la intervención médico-científica^4^^,^^5^, las negociaciones y tensiones con las comunidades religiosas^6^^,^^7^^,^^8^^,^^9^^,^^10^ y el papel que ocupan tanto el Estado como el mercado en la configuración de políticas sexuales y reproductivas^11^^,^^12^. La salud reproductiva, en tanto campo de disputas, no se reduce a la esfera biomédica: constituye un terreno en el que se negocian derechos, identidades, proyectos de vida y sentidos colectivos.

Desde esta perspectiva, los artículos que conforman el dossier “Salud reproductiva: intersecciones entre tecnología y reproducción”, publicado en *Salud Colectiva*, proponen abordar ese cruce complejo y multifacético entre tecnologías y reproducción, subrayando un punto de vista particular: el de las intersecciones. Nos interesa detenernos en ese “entre”, porque allí se condensan experiencias, tensiones y contradicciones que iluminan la salud colectiva en su dimensión más amplia y que muestran cómo distintos registros -tecnológicos, jurídicos, afectivos y morales- se articulan de manera situada. Así, los avances científicos trascienden su rol instrumental y se presentan como actores políticos capaces de transformar cuerpos, relaciones y futuros posibles.

En este marco, una de las preguntas que orienta las indagaciones es: ¿de qué manera estas transformaciones, lejos de desplegarse de forma homogénea, se moldean y encuentran resistencias en regímenes normativos, historias nacionales, matrices religiosas y dispositivos regulatorios específicos? Retomando a Charis Thompson^13^, entendemos las coreografías reproductivas como interacciones co-constitutivas entre entidades humanas (gestantes, familias, donantes, profesionales de la salud) y entidades no humanas (laboratorios, ecografías, fármacos, técnicas quirúrgicas). Desde esta clave analítica, el dossier avanza en reconocer que la reproducción nunca es un hecho meramente biológico, sino el resultado de un ensamblaje complejo de actores, objetos, discursos y emociones. 

En esta clave, este editorial busca también habilitar una lectura transnacional. Enmarcadas en Argentina, Brasil y España, nuestras investigaciones permiten observar cómo diferentes historias repronacionales -atravesadas por regímenes políticos, matrices religiosas, modelos de bienestar y dinámicas de mercado particulares- iluminan tanto contrastes como resonancias comunes. En las secciones que siguen, el diálogo entre estos tres contextos nacionales permitirá explorar cómo tecnologías, políticas y experiencias se entrecruzan de manera situada, produciendo configuraciones específicas y, a la vez, dinámicas compartidas en el campo de la salud reproductiva.

## Tecnologías reproductivas

El impacto de las tecnologías en el campo de la sexualidad y la reproducción es quizás uno de los fenómenos más reveladores del modo en que la ciencia y la técnica inciden en las formas de vida contemporáneas. Lejos de limitarse a una cuestión instrumental, las tecnologías reproductivas han transformado los imaginarios sociales, las prácticas íntimas y las posibilidades de acción individual y colectiva.

La introducción y masificación de métodos anticonceptivos constituyen un ejemplo paradigmático. La píldora anticonceptiva, en particular, no solo modificó las condiciones materiales de posibilidad de la sexualidad, sino que encarnó un emblema de autonomía y modernidad. Sin embargo, como muestra Isabella Cosse^14^, en la Argentina urbana de la década de 1960, este proceso no se tradujo en una revolución como tal, sino en lo que ella denomina “revoluciones discretas”: transformaciones en la sexualidad y la reproducción que presentan continuidades, rupturas con mandatos y modelos de familia. Este matiz recuerda que la tecnología no actúa en abstracto, sino que se inserta en contextos históricos y socioculturales específicos.

La historia repronacional argentina también evidencia el rol central del Estado en la gobernanza de la reproducción. En Argentina, a lo largo del siglo XX, el Estado intervino en un doble registro: la reducción de la mortalidad infantil, por un lado, y el control de la natalidad, por otro, como documenta el trabajo de Marcela Nari^15^. Esta doble operación ilustra el modo en que la ideología maternal y la tutela estatal conformaron un entramado disciplinario sobre los cuerpos de las mujeres y personas gestantes.

El caso de España destaca por haber pasado de un “*baby boom*” a una “infertilidad estructural” en cuatro décadas, generando intensos debates y cuestionamientos sobre nuevas formas de concebir la maternidad^16^. Así, tal y como ha sucedido en otros países europeos, la historia repronacional en España ha estado marcada por varios condicionantes sociopolíticos que han generado un retraso en la maternidad y un declive en la fertilidad^16^. Entre otras cuestiones, la despenalización del aborto, el uso extensivo de preservativos y el acceso de las mujeres a la educación y al mundo laboral, junto con la falta de políticas sociales que favorezcan la natalidad y la conciliación, han situado España como uno de los países europeos con natalidad más baja^17^^,^^18^. Actualmente, la tasa de fecundidad en España es de 1,12^19^, confirmando una tendencia de descenso continuado desde 2017.

Por su parte, la fecundidad en Brasil ha ido disminuyendo desde mediados de la década de 1960, momento en el que las mujeres tenían, en promedio, alrededor de seis hijos. En ese entonces, debido a la dificultad para obtener métodos anticonceptivos eficaces, recurrían con frecuencia a la esterilización mediante ligadura de trompas y al aborto. En 1986, el 44% de las brasileñas en edad fértil estaban esterilizadas. Entre 1991 y 2000, la caída de la fecundidad fue más pronunciada entre las mujeres con menor nivel educativo, en situación socioeconómica más vulnerable, negras o residentes en zonas rurales. Como resultado, en 2015 la tasa nacional se ubicó aproximadamente en 1,72 hijos por mujer, es decir, por debajo de la tasa de reemplazo. El censo demográfico de 2010 registró una caída en la fecundidad entre mujeres de 15 a 19 años, lo cual sugiere un posible aplazamiento en el inicio de la reproducción, en paralelo con un aumento significativo de la escolaridad femenina^20^. El descenso de la fecundidad también está asociado al aumento significativo de la cobertura del acceso a los anticonceptivos desde finales de la década de 1980^20^. Los datos del último censo demográfico de 2022 muestran la continuidad de la tendencia a la baja de la fecundidad: las mujeres brasileñas están teniendo menos hijos y posponiendo cada vez más la maternidad. También ha aumentado considerablemente la proporción de mujeres que llegan al final de su vida reproductiva sin haber tenido hijos. En 1960, la tasa de fecundidad total del país era de 6,28 hijos por mujer. En 2022, esta tasa se redujo a 1,55 hijos por mujer^21^. 

En el presente, las tecnologías reproductivas han abierto un horizonte novedoso en la construcción de parentescos y familias. La multiplicación de agentes involucrados -comitentes, gestantes, donantes- ha puesto en cuestión la naturalización de la heteronorma y la biparentalidad, desafiando los modelos tradicionales de filiación y habilitando la emergencia de constelaciones familiares alternativas. 

Un panorama de estas dinámicas se refleja en las tasas de utilización de las técnicas de reproducción humana asistida (TRHA). De acuerdo con el Registro Latinoamericano de Reproducción Asistida (RLA) de 2021, en Argentina, se reportaron 21.379 ciclos de fertilización -que representan una tasa de 480,2 ciclos de fertilización por millón de habitantes-, mientras que en Brasil fueron 56.317 los ciclos de fertilización para el mismo período. En términos de nacimientos, aproximadamente el 0,63% de los niños nacidos vivos en Argentina provienen de técnicas de reproducción asistida, frente al 0,35 % en Brasil^22^. Dentro del reporte se releva un amplio abanico de técnicas que incluyen, además de la preservación de la fertilidad, ciclos con gametos propios recién obtenidos o con embriones criopreservados y distintos procedimientos basados en la donación de gametos. También ha aumentado la visibilidad y la demanda de gestación por subrogación^23^. 

En el caso de España, según el Registro Nacional de Actividad de la Sociedad Española de Fertilidad, en 2022 se realizaron 167.195 ciclos de fecundación in vitro y 31.635 inseminaciones artificiales, que representan un 12% de los nacimientos en España^24^. Estos datos muestran que el uso de tratamientos de reproducción se ha mantenido en los últimos años, después de que en 2021 hubiera más tratamientos de los esperados por la pandemia de covid-19. Además, el último registro destaca la popularidad y el éxito creciente de la vitrificación de ovocitos como técnica de preservación de la fertilidad y con tasas elevadas de éxito. España, además, se ha convertido en uno de los destinos principales para técnicas de reproducción asistida, siendo las mujeres francesas las principales usuarias de origen extranjero^17^. 

En lo que respecta a las técnicas de reproducción asistida del contexto brasileño, un aspecto destacable es la desigualdad en el acceso. Un estudio realizado por el Fondo de Población de las Naciones Unidas en 2024 muestra que la reproducción asistida se concentra principalmente en el sector privado y que, a pesar de algunos intentos, nunca se ha convertido en una prioridad dentro del sistema público de salud. De este modo, se evidencia una contradicción entre la disponibilidad de técnicas innovadoras y las posibilidades reales de acceso: en la práctica, estos servicios quedan restringidos a quienes pueden costearlos^25^. 

Esta tensión entre marco normativo y acceso material también se manifiesta en el caso argentino, aunque con otros matices. Si bien, tal como veremos, Argentina cuenta con una ley que garantiza la cobertura universal de las técnicas de reproducción humana asistida, su implementación ha sido afectada por limitaciones económicas que impactan directamente en la utilización: entre 2019 y 2021, los ciclos completos de tratamiento por millón de habitantes disminuyeron, evidenciando que la gratuidad formal no siempre se traduce en accesibilidad efectiva^22^. Así, ambos contextos permiten pensar cómo las políticas reproductivas se inscriben en economías afectivas y materiales que inciden en el ejercicio de la autonomía reproductiva. 

En vista de estos procesos históricos, locales y situados, se advierte que las tecnologías no solo transforman prácticas íntimas, sino que disputan sentidos sociales y culturales más amplios, en torno a la legitimidad de los vínculos y el reconocimiento de derechos articulando dimensiones técnicas, legales y simbólicas en procesos que exceden la clínica. 

## Salud reproductiva

Si las tecnologías permiten abrir nuevas posibilidades, el campo de la salud reproductiva invita a pensar cómo esas posibilidades se inscriben en políticas, servicios y prácticas concretas. Aquí emergen tensiones entre acceso formal, implementación efectiva y calidad de la atención.

Entre estos distintos niveles, el Estado ocupa una posición estratégica: lejos de ser un mero garante, interviene activamente en la gobernanza de las personas usuarias, gestantes, donantes, profesionales, así como de los embriones y sustancias biológicas; en la definición de los alcances de la autonomía como en la regulación de las prácticas biomédicas y en la administración de derechos. Así, las políticas públicas en torno a las tecnologías reproductivas se constituyen en un espacio donde confluyen saberes científicos, marcos jurídicos y disputas morales, y donde se dirime qué formas de familia y filiación resultan socialmente legítimas^3^^,^^12^.

Con la primera Ley de Reproducción Humana Asistida en 1988, España se convirtió en uno de los primeros países europeos en regular estas técnicas. Sin embargo, fue con la ley de 2006 -actualmente en vigor- cuando se reconoció el acceso universal a estos tratamientos a todas las mujeres sin distinguir su estado civil y su orientación sexual. Así, España se convirtió en un país pionero en materia de salud reproductiva. Además, las técnicas fueron introducidas en la cobertura del Sistema Nacional de Salud para poder garantizar el acceso universal a todo el territorio. El avance legal más reciente en materia reproductiva en España ha sido la llamada “Ley trans” (Ley 4/2023) en la que se incluyó el reconocimiento de “las personas trans con capacidad de gestar, sin discriminación por motivos de identidad sexual”, y el acceso a todos los servicios y derechos reproductivos garantizados por el sistema nacional de salud. 

Sin embargo, uno de los retos principales del actual marco legal en España es la organización descentralizada de las competencias en salud en el territorio, que supone que, en la práctica, el acceso a los derechos reproductivos puede ser diferente en función de la comunidad autónoma en la que habiten las mujeres. 

Las técnicas de reproducción humana asistida en España son una alternativa atractiva ante la situación de ilegalidad de la gestación subrogada y las dificultades encontradas para adoptar^26^. Sin embargo, ambas prácticas están presentes, lo que genera debates relevantes en torno a la labor reproductiva, el anonimato y el altruismo^26^^,^^27^. España se caracteriza por garantizar el anonimato de los donantes de gametos y, por consiguiente, por su alta disponibilidad de ovocitos y material donado para las técnicas de reproducción humana asistida. Así, el marco legal ha favorecido el creciente mercado reproductivo en España, destino principal para las “movilidades reproductivas”.

En Argentina, un conjunto de legislaciones garantiza formalmente el acceso a las técnicas de reproducción humana asistida. Estas técnicas se practican desde hace más de treinta años en el ámbito privado. Previo a la sanción de la Ley de Reproducción Humana Asistida (Ley 26862, 2013), eran las asociaciones de medicina reproductiva las que regulaban el campo de las técnicas. Asimismo, durante este período, el acceso era restringido, por tratarse de un conjunto de tratamientos onerosos para la mayoría de la población. La sanción de la ley en 2013 significó un punto de inflexión: estableció la cobertura a nivel nacional, abrió centros públicos especializados y despatologizó su acceso, previamente ligado a la infertilidad. Esta decisión implicó un cambio profundo en la política de salud: el foco pasó de la familia heterosexual nuclear hacia la habilitación del acceso gratuito a toda persona mayor de edad, cualquiera que sea su orientación sexual o estado civil y tipo de cobertura de salud.

Posteriormente, en 2015, el Código Civil y Comercial incorporó la filiación de las personas nacidas mediante tratamientos de reproducción asistida, reconociendo la “voluntad procreacional”, entendida como la decisión libre de asumir la maternidad o paternidad independientemente del origen de los gametos y más allá del vínculo biológico (Ley 26994, 2015, v. 562)^8^^,^^28^. Esta reforma permitió adaptar el marco jurídico a las innovaciones científicas introducidas por las técnicas de reproducción humana asistida y a la diversidad de conformaciones familiares que posibilitan. Así, el reconocimiento legal consolidó un marco normativo que amplió los márgenes de legitimidad de las nuevas formas de familia.

En lo que respecta a Brasil, existe un vacío normativo. La reproducción médicamente asistida está contemplada en el Código Civil brasileño de 2002 únicamente en lo relativo a regular las relaciones de filiación, reconociendo a los hijos concebidos mediante reproducción asistida^29^, incluyendo los casos de donación de gametos y embriones, así como la reproducción póstuma, siempre que haya sido autorizada en vida. También existe una disposición del Consejo Nacional de Justicia sobre la emisión de certificados de nacimiento, que reconoce el registro de los ascendientes en casos de unión homoafectiva y excluye el nombre de la parturienta en casos de gestación subrogada. La misma disposición no reconoce el vínculo de parentesco del donante en caso de reproducción asistida. De este modo, la reproducción asistida en Brasil está regulada principalmente por resoluciones del Consejo Federal de Medicina (CFM). La evolución de estas resoluciones varía según los cambios en la sociedad y también del contexto político. Así, en la primera resolución (1358/92), la reproducción asistida se aplicaba únicamente a parejas infértiles y mujeres solteras. Con el paso de los años, se incluyeron las parejas homosexuales, ampliando el acceso a la gestación subrogada y permitiendo la recepción de óvulos de la pareja^25^. Hasta la resolución de 2021, era obligatorio el anonimato en la donación de gametos. Luego se admitió una excepción: la donación de gametos y embriones entre personas con parentesco consanguíneo hasta el cuarto grado con el receptor. No se acepta la gestación subrogada comercial, pero la gestante subrogada debe pertenecer a la familia de uno de los miembros de la pareja en parentesco consanguíneo hasta el cuarto grado. 

Aun con la actualización de los marcos normativos en Argentina, Brasil y España, persisten vacíos legales que dan lugar a debates públicos y litigios judiciales, reabriendo interrogantes éticos y políticos sobre distintas dimensiones asociadas a las tecnologías reproductivas. En Argentina, la falta de regulaciones claras en áreas como la gestación por sustitución produce incertidumbre jurídica y controversias sobre su legitimidad, mientras que el estatuto de la vida embrionaria y la definición de quiénes son los actores legítimos para decidir su destino siguen siendo objeto de debate^28^^,^^30^. En Brasil, las resoluciones sobre embriones han oscilado con el tiempo: tras permitir únicamente la criopreservación y la donación a otras parejas, con el paso de los años se autorizó el descarte en ciertas situaciones; sin embargo, en 2021 y 2022 se restableció la protección total de los embriones, en consonancia con la orientación conservadora del Consejo Federal de Medicina y su alineamiento con el Gobierno de Bolsonaro, evidenciando la influencia de contextos políticos en la regulación^3^^,^^29^. En España, persiste un debate sobre el marco legal y ético del anonimato, impulsado en 2022 por la creación de la agrupación de personas donoconcebidas Asociación de Hijas e Hijos de Donantes (AHID). Además, según Lafuente Funes^11^, el mercado reproductivo en España está creciendo en un contexto de desigualdades globales, generando dilemas complejos sobre la bioeconomía reproductiva que se activa en estos mercados. Aunque este análisis se centra en España, consideramos que estas dinámicas son también relevantes para Argentina y Brasil, donde los mercados reproductivos presentan tensiones similares y plantean interrogantes análogos sobre las consecuencias sociales, éticas y económicas de los avances tecnológicos en reproducción asistida.

Al mismo tiempo, la accesibilidad formal se presenta como condición necesaria pero no suficiente. Si bien, tal como advertimos, existen marcos regulatorios y limitaciones, la experiencia cotidiana muestra resistencias persistentes: la ligadura tubaria, por ejemplo, si bien es reconocida legalmente en la Argentina, continúa sujeta a restricciones arbitrarias que limitan el acceso real de las usuarias del sistema de salud, tal como analiza el trabajo de Galende Villavicencio que compone este dossier^31^.

La legalización del aborto constituye, sin dudas, un hito que reconfiguró tanto las políticas públicas como las culturas de atención, de manera muy desigual según el país. En España, la interrupción voluntaria del embarazo es legal desde 2010, consolidando un marco regulatorio amplio y generando cambios significativos en los servicios de salud y en la percepción social del derecho a decidir. En Argentina, la legalización en 2020 marcó un avance histórico, reforzando los derechos reproductivos en un contexto de fuerte activismo feminista. En contraste, Brasil mantiene una normativa altamente restrictiva: el Código Penal de 1940 solo permite el aborto en dos situaciones específicas -cuando no existe otro medio para salvar la vida de la mujer y en casos de violencia sexual-, lo que refleja un contexto político y cultural donde la criminalización sigue limitando profundamente el acceso y la autonomía reproductiva de las mujeres. En 2012, una decisión del Tribunal Supremo Federal pasó a permitir el aborto en caso de anencefalia. A pesar de la ilegalidad del aborto en Brasil, las investigaciones muestran que la práctica es muy habitual. Una encuesta nacional realizada en zonas urbanas en 2010, y repetida en 2016, revela que casi una de cada cinco mujeres brasileñas de 40 años ha abortado al menos una vez^32^. La ilegalidad del aborto no impide que se practique, pero acentúa las desigualdades sociales: las mujeres en mejores condiciones socioeconómicas pagan por el aborto en clínicas privadas, mientras que la mayoría recurre al misoprostol y, al inicio del sangrado, acude al servicio público para realizar allí el vaciamiento uterino, generalmente por legrado, y tratar las complicaciones^20^.

Una acción en curso en el Tribunal Supremo Federal solicita la despenalización del aborto voluntario hasta las 12 semanas^33^. En 2018, se celebró una audiencia pública y solo hubo un voto favorable de la ponente de la acción en 2023. Esta iniciativa encuentra una fuerte resistencia en el Congreso Nacional, cuya composición mayoritaria es conservadora, además de la resistencia del actor más importante, la Iglesia Católica, a la que se asocian segmentos religiosos conservadores, especialmente evangélicos y espiritistas kardecistas. El debate público sobre el aborto es incipiente. 

Sin embargo, para el caso argentino, su incorporación efectiva revela todavía un mapa desigual, atravesado por resistencias institucionales, diferencias regionales y tensiones entre discursos normativos y prácticas concretas. En este sentido, el aporte de Briana Keefe-Oates y su equipo^34^ resulta clave: su trabajo en la construcción de cuestionarios para evaluar políticas públicas permite producir datos situados, en clave feminista, que visibilizan cómo se materializan -o no- los derechos en contextos locales específicos. Estas dificultades también están presentes en otros contextos en los que, a pesar del marco legal, sigue habiendo una estigmatización de la interrupción voluntaria del embarazo^35^.

Por otra parte, la introducción de tecnologías farmacológicas como la mifepristona, avalada por la Administración Nacional de Medicamentos, Alimentos y Tecnología Médica de Argentina, y recomendada por la OMS, muestra de qué modo la innovación técnica incide en la ampliación de derechos y en la configuración de la salud colectiva. El medicamento no es solo una herramienta terapéutica: es también un actor político que modifica accesos, redefine itinerarios de atención y reconfigura los horizontes de la experiencia de la reproducción.

Los distintos procesos aquí mencionados -desde la ligadura tubaria y el aborto hasta la gestación por sustitución y la incorporación de tecnologías farmacológicas- muestran que la salud reproductiva se configura en un terreno de ensamblajes humanos y no humanos y está atravesado por disputas legales, institucionales y culturales. Lejos de tratarse de un campo homogéneo, lo que emerge es una trama de prácticas, regulaciones y experiencias que se ensamblan de manera situada, de acuerdo a las historias repronacionales y los diálogos transnacionales. 

## Intersecciones

Más allá de analizar separadamente la dimensión tecnológica y la dimensión sanitaria, este dossier busca explorar las intersecciones: esos espacios donde actores humanos y no humanos se articulan en redes de cooperación, conflicto y negociación, produciendo configuraciones inéditas de experiencia y de sentido. Los artículos que componen este dossier enfocan distintos aspectos y dimensiones de estas intersecciones.

El aborto medicamentoso constituye un ejemplo privilegiado. Antes de su legalización, la circulación de misoprostol y mifepristona en redes feministas y profesionales permitió la constitución de prácticas seguras y menos estigmatizadas. Como muestran Lenta y Longo^36^, la medicación adquirió una agencia propia: se volvió productora de abortos posibles, configurando subjetividades y trayectorias colectivas que desafiaron el estigma y habilitaron nuevas formas de cuidado. Aquí, la tecnología aparece no solo como instrumento, sino como dispositivo con capacidad de acción dentro de un entramado político y cultural más amplio.

La gestación por sustitución ofrece otro caso de intersección reveladora. El artículo de Ariza y Lima^37^ sobre las relaciones entre mujeres cisheterosexuales casadas que buscan la maternidad mediante gestantes ucranianas muestra cómo este proceso excede el marco contractual para convertirse en una red distribuida y colaborativa. Las autoras, siguiendo la noción de enredos reproductivos de Anika König -inspirada en Rayna Rapp-, señalan que allí confluyen actores, objetos y tecnologías que operan como dispositivos de vinculación, produciendo relaciones sociales y afectivas imposibles de reducir a una dimensión puramente biomédica o jurídica.

En ambos casos se evidencia que las tecnologías reproductivas no median simplemente entre sujetos y fines, sino que actúan como elementos constitutivos de nuevas realidades. Son, en sí mismas, productoras de subjetividades, parentescos y sentidos colectivos.

El recorrido por estas tres dimensiones -tecnologías, salud e intersecciones- permite comprender que la reproducción nunca es un hecho natural, dado o universal. Es, en cambio, el resultado de ensamblajes situados, atravesados por regímenes de saber y poder, por normativas estatales y dinámicas de mercado, por discursos culturales y prácticas de resistencia. Pensar las tecnologías reproductivas y la salud reproductiva en clave interseccional nos invita a reconocer que lo que está en juego no son solo procedimientos biomédicos, sino la definición misma de cuerpos, vínculos y futuros posibles.

Finalmente, las editoras invitadas para esta convocatoria queremos agradecer a las autoras, al comité editorial de la revista *Salud Colectiva* y a las y los revisores externos que evaluaron los artículos de este dossier. Este esfuerzo colectivo ha hecho posible reunir trabajos que, esperamos, contribuyan a comprender la reproducción como un entramado complejo de actores, objetos, discursos y emociones, más allá de su reducción a un hecho meramente biológico o natural. Con esta mirada buscamos aportar herramientas para analizar y afrontar críticamente los desafíos que la salud reproductiva plantea hoy a nuestras sociedades.
