# Supplementary material for: Salud reproductiva: intersecciones entre tecnología y reproducción
Source: Salud Colect. 2025 Oct 3;21:e5953. [Article in Spanish] doi: 10.18294/sc.2025.5953 (PMC12724426; doi:10.18294/sc.2025.5953)
Supplement: Translation

# Translation: Reproductive health: Intersections between technology and reproduction

**Authors:** Ana Lucía Olmos Álvarez, María Cecilia Johnson, Naara Luna, Rosa Martínez-Cuadros

**Affiliations:** 1 Doctor of Social Anthropology. Assistant researcher, Consejo Nacional de Investigaciones Científicas y Técnicas, based at the Universidad Nacional de Avellaneda, Buenos Aires, Argentina. analuciaolmos@gmail.com; 2 Doctor in Gender Studies. Assistant researcher, Consejo Nacional de Investigaciones Científicas y Técnicas, based at the Center for Research and Studies on Culture and Society, Universidad Nacional de Córdoba, Córdoba, Argentina. cecilia.johnson@unc.edu.ar; 3 Doctor of Anthropology. Associate Professor, Universidade Federal Rural do Rio de Janeiro, Brazil. naaraluna2015@gmail.com; 4 Doctor of Sociology. Postdoctoral researcher, GENI group, Universitat de Barcelona, Barcelona, Spain. rosamartinezcuadros@gmail.com

## Introduction

In a century marked by rapid technological innovation and profound social transformations, reproductive health has emerged as one of the most dynamic and contested fields, reshaping not only individual intimacy but also family structures, citizenship rights, and state policies on a global scale. The conjunction of scientific developments and broader public access to certain practices has opened up unforeseen horizons regarding sexuality, reproduction, and parenthood projects. However, far from constituting a homogeneous or universal process, these changes are traversed by normative regimes, national histories, religious and moral frameworks, and regulatory mechanisms that configure distinct conditions of possibility. In this sense, and following the proposal of Sarah Franklin and Marcia Inhorn,^1^ we may speak of repronational histories: specific assemblages in which technologies, bodies, and politics acquire particular - and often conflicting - meanings.

Reproductive technologies have enabled the emergence of experiences and forms of agency that, for a long time, remained invisible. At the same time, they have raised unavoidable questions about the multiple meanings and forms of recognition of kinship ties,^2^^,^^3^ the ethical and social dilemmas surrounding the limits of medical-scientific intervention,^4^^,^^5^ the negotiations and tensions with religious communities,^6^^,^^7^^,^^8^^,^^9^^,^^10^ and the roles played by both the state and the market in shaping sexual and reproductive policies.^11^^,^^12^ Reproductive health, as a field of dispute, cannot be reduced to the biomedical sphere: it constitutes a terrain where rights, identities, life projects, and collective meanings are continuously negotiated.

From this perspective, the articles gathered in the dossier *“Reproductive Health: Intersections between Technology and Reproduction,”* published in *Salud Colectiva*, address this complex and multifaceted interplay between technologies and reproduction, highlighting a particular standpoint: that of the intersections. We are interested in dwelling on this “in-between,” for it is there that experiences, tensions, and contradictions converge illuminating collective health in its broadest sense and revealing how different dimensions - technological, legal, affective, and moral - are articulated in situated ways. In this light, scientific advances transcend their instrumental role and emerge as political actors capable of transforming bodies, relationships, and possible futures.

Within this framework, one of the guiding questions of these inquiries is: how do these transformations - far from unfolding uniformly - take shape and encounter resistance within specific normative regimes, national histories, religious matrices, and regulatory dispositifs? Drawing on Charis Thompson,^13^ we understand reproductive choreographies as co-constitutive interactions between human entities (gestating individuals, families, donors, health professionals) and non-human entities (laboratories, ultrasounds, pharmaceuticals, surgical techniques). From this analytical standpoint, the dossier underscores that reproduction is never merely a biological fact, but rather the outcome of a complex assemblage of actors, objects, discourses, and emotions.

In this key, this editorial also seeks to enable a transnational reading. Framed in Argentina, Brazil, and Spain, our research reveal how different repronational histories - shaped by specific political regimes, religious matrices, welfare models, and market dynamics - shed light on both contrasts and shared resonances. In the following sections, the dialogue among these three national contexts allows us to explore how technologies, policies, and experiences intersect in situated ways, producing specific configurations while also generating shared dynamics within the field of reproductive health.

## Reproductive technologies

The impact of technologies in the field of sexuality and reproduction is perhaps one of the most revealing phenomena of how science and technology shape contemporary forms of life. Far from being a merely instrumental matter, reproductive technologies have transformed social imaginaries, intimate practices, and the possibilities for both individual and collective agency.

The introduction and widespread use of contraceptive methods constitute a paradigmatic example. The contraceptive pill, in particular, not only modified the material conditions of possibility for sexuality but also came to embody an emblem of autonomy and modernity. However, as Isabella Cosse^14^^)^ shows, in urban Argentina during the 1960s this process did not translate into a revolution per se, but rather into what she calls “discreet revolutions”: transformations in sexuality and reproduction that combine continuities and ruptures with traditional mandates and family models. This nuance reminds us that technology does not operate in the abstract, but rather becomes embedded within specific historical and sociocultural contexts.

Argentina’s repronational history also highlights the central role of the state in the governance of reproduction. Throughout the twentieth century, the Argentine state intervened on two fronts: reducing infant mortality, on the one hand, and controlling birth rates, on the other, as documented by Marcela Nari.^15^ This dual operation illustrates how maternal ideology and state tutelage together constituted a disciplinary framework over the bodies of women and gestating individuals.

The case of Spain stands out for having moved from a “baby boom” to a situation of “structural infertility” within four decades, sparking intense debates and questions about new ways of conceiving motherhood.^16^ As in other European countries, Spain’s repronational history has been shaped by various sociopolitical factors that have led to delayed motherhood and a decline in fertility.^16^ Among these factors are the decriminalization of abortion, the extensive use of condoms, women’s access to education and the labor market, and the absence of social policies that promote childbirth and work-family balance all of which have placed Spain among the European countries with the lowest birth rates.^17^^,^^18^ Currently, Spain’s fertility rate stands at 1.12,^19^ confirming a continued downward trend since 2017.

In Brazil, fertility has been declining since the mid-1960s, when women had an average of around six children. At that time, due to limited access to effective contraceptive methods, women frequently resorted to sterilization through tubal ligation and to abortion. By 1986, 44% of Brazilian women of reproductive age were sterilized. Between 1991 and 2000, the drop in fertility was most pronounced among women with lower levels of education, in more vulnerable socioeconomic situations, Black women, and those living in rural areas. As a result, by 2015 the national fertility rate was approximately 1.72 children per woman, below the replacement level. The 2010 demographic census recorded a decrease in fertility among women aged 15 to 19, suggesting a possible postponement of the onset of reproduction, alongside a significant increase in women’s educational attainment.^20^ The decline in fertility is also associated with the marked expansion of access to contraceptives since the late 1980s.^20^ Data from the most recent 2022 demographic census confirm the continued downward trend in fertility: Brazilian women are having fewer children and increasingly postponing motherhood. The proportion of women reaching the end of their reproductive lives without having had children has also risen significantly. In 1960, the country’s total fertility rate was 6.28 children per woman; by 2022, this rate had fallen to 1.55.^21^

Today, reproductive technologies have opened up new horizons in the construction of kinship and family. The multiplication of actors involved - intended parents, gestating individuals, donors - has challenged the naturalization of heteronormativity and biparenthood, questioning traditional models of filiation and enabling the emergence of alternative family constellations.

A snapshot of these dynamics can be observed in the rates of use of assisted reproductive technologies (ART). According to the 2021 Latin American Registry of Assisted Reproduction (RLA), Argentina reported 21,379 fertilization cycles - representing a rate of 480.2 fertilization cycles per million inhabitants - while Brazil reported 56,317 cycles during the same period. In terms of births, approximately 0.63% of live-born children in Argentina resulted from assisted reproduction techniques, compared with 0.35% in Brazil.^22^ The report includes a wide range of procedures, encompassing fertility preservation, cycles using recently obtained own gametes or cryopreserved embryos, and various methods involving gamete donation. The visibility and demand for gestational surrogacy have also increased.^23^

In Spain, according to the National Activity Registry of the Spanish Fertility Society, 167,195 in vitro fertilization (IVF) cycles and 31,635 artificial inseminations were performed in 2022, accounting for 12% of all births in the country.^24^ These data show that the use of reproductive treatments has remained stable in recent years, following a temporary rise in 2021 linked to procedures postponed during the COVID-19 pandemic. The most recent report also highlights the growing popularity and success of oocyte vitrification as a fertility preservation technique, with high success rates. Furthermore, Spain has become one of the main international destinations for assisted reproduction, with French women constituting the largest group of foreign users.^17^

In the Brazilian context, a key aspect of assisted reproductive technologies is the inequality of access. A 2024 study by the United Nations Population Fund (UNFPA) shows that assisted reproduction is concentrated mainly in the private sector and, despite some efforts, has never become a priority within the public health system. This reveals a contradiction between the availability of innovative techniques and the real possibilities of access: in practice, these services remain limited to those who can afford them.^25^

This tension between the regulatory framework and material access is also evident in the Argentine case, albeit with different nuances. Although, as we shall see, Argentina has a law that guarantees universal coverage for ART, its implementation has been affected by economic constraints that directly impact their use: between 2019 and 2021, the number of complete treatment cycles per million inhabitants declined, revealing that formal gratuity does not always translate into effective accessibility.^22^ Both contexts thus invite reflection on how reproductive policies are inscribed within affective and material economies that shape the exercise of reproductive autonomy.

In light of these historical, local, and situated processes, it becomes clear that technologies not only transform intimate practices but also contest broader social and cultural meanings related to the legitimacy of relationships and the recognition of rights articulating technical, legal, and symbolic dimensions in processes that extend beyond the clinical sphere.

## Reproductive health

If technologies open up new possibilities, the field of reproductive health invites reflection on how these possibilities become embedded in concrete policies, services, and practices. Here, tensions emerge between formal access, effective implementation, and quality of care.

Across these different levels, the state occupies a strategic position: far from being a mere guarantor, it actively intervenes in the governance of users, gestating persons, donors, and professionals, as well as embryos and biological substances. It defines the scope of autonomy, regulates biomedical practices, and administers rights. Thus, public policies surrounding reproductive technologies constitute a space where scientific knowledge, legal frameworks, and moral disputes converge, and where the social legitimacy of certain forms of family and filiation is negotiated.^3^^,^^12^

With the first Assisted Human Reproduction Law enacted in 1988, Spain became one of the first European countries to regulate these technologies. However, it was with the 2006 law - still in force today - that universal access to these treatments was recognized for all women, regardless of marital status or sexual orientation. Spain became a pioneering country in the field of reproductive health. In addition, these techniques were incorporated into the coverage of the National Health System in order to ensure universal access across the country.

The most recent legal advancement in reproductive matters in Spain is the so-called “Trans Law” (Law 4/2023), which includes the recognition of “trans persons with the capacity to gestate, without discrimination based on sexual identity,” and guarantees their access to all reproductive services and rights provided by the national health system.

However, one of the main challenges of the current legal framework in Spain lies in the decentralized organization of health competences across the territory, which means that, in practice, access to reproductive rights may vary depending on the autonomous community in which women reside.

Assisted reproductive technologies in Spain constitute an attractive alternative in the face of the illegality of surrogacy and the difficulties encountered in adoption.^26^ Nonetheless, both practices are present, giving rise to significant debates around reproductive labor, anonymity, and altruism.^26^^,^^27^ Spain is characterized by its legal guarantee of gamete donor anonymity and, consequently, by a high availability of oocytes and donated material for assisted reproductive procedures. This legal framework has thus fostered the growth of the reproductive market in Spain, positioning the country as a primary destination for “reproductive mobilities.”

In Argentina, a set of laws formally guarantees access to ART. These techniques have been practiced for more than thirty years in the private sector. Prior to the enactment of the Assisted Human Reproduction Law (Law 26.862, 2013), it was the reproductive medicine associations that regulated the field. During this period, access was restricted, as these treatments were costly and therefore inaccessible to most of the population. The passage of the 2013 law marked a turning point: it established nationwide coverage, created specialized public centers, and depathologized access, which had previously been tied to infertility. This decision entailed a profound shift in health policy: from focusing on the heterosexual nuclear family to guaranteeing free access for any adult, regardless of sexual orientation, marital status, or type of health coverage.

Subsequently, in 2015, the Civil and Commercial Code incorporated the filiation of persons born through assisted reproductive treatments, recognizing the notion of “procreative will,” understood as the free decision to assume motherhood or fatherhood independently of the origin of the gametes and beyond biological ties (Law 26.994, 2015, v. 562).^8^^,^^28^ This reform allowed the legal framework to adapt to the scientific innovations introduced by assisted reproductive technologies and to the diversity of family configurations they make possible. In doing so, legal recognition consolidated a normative framework that expanded the boundaries of legitimacy for new forms of family.

In Brazil, there is a regulatory vacuum. Medically assisted reproduction is included in the 2002 Civil Code only insofar as it regulates filiation relations, recognizing children conceived through assisted reproduction,^29^ including cases involving gamete and embryo donation, as well as posthumous reproduction, provided it was authorized while alive. There is also a provision from the National Council of Justice regarding the issuance of birth certificates, which recognizes the registration of parents in cases of same-sex unions and omits the name of the birth mother in cases of surrogacy. The same provision does not recognize kinship ties between donors and children conceived through assisted reproduction. Consequently, assisted reproduction in Brazil is regulated mainly through resolutions of the Federal Council of Medicine (CFM). The evolution of these resolutions has varied according to social change and political context. The first resolution (1358/92) limited assisted reproduction to infertile couples and single women. Over the years, access was extended to same-sex couples, broadening eligibility for surrogacy and allowing for egg donation between partners.^25^ Until the 2021 resolution, gamete donation anonymity was mandatory; an exception was then introduced, permitting gamete and embryo donation between persons related by consanguinity up to the fourth degree with the recipient. Commercial surrogacy remains prohibited, but the surrogate must belong to the family of one of the partners, also within the fourth degree of consanguinity.

Even with the updating of regulatory frameworks in Argentina, Brazil, and Spain, legal gaps persist, giving rise to public debate and judicial litigation that reopen ethical and political questions about various dimensions associated with reproductive technologies. In Argentina, the absence of clear regulations in areas such as surrogacy generates legal uncertainty and controversy over its legitimacy, while the status of embryonic life and the question of who holds legitimate authority to determine its fate remain contested.^28^^,^^30^ In Brazil, the resolutions concerning embryos have fluctuated over time: after initially allowing only cryopreservation and donation to other couples, later updates authorized disposal in certain situations; however, in 2021 and 2022, total protection of embryos was reinstated, in line with the conservative orientation of the Federal Council of Medicine and its alignment with the Bolsonaro administration, revealing the influence of political contexts on regulation.^3^^,^^29^ In Spain, debate persists regarding the legal and ethical framework surrounding anonymity, particularly since 2022, when the Association of Donor-Conceived Daughters and Sons (AHID) was founded. Moreover, according to Lafuente Funes,^11^ the reproductive market in Spain continues to expand within a context of global inequalities, generating complex dilemmas around the reproductive bioeconomy activated in these markets. Although this analysis focuses on Spain, we consider these dynamics equally relevant to Argentina and Brazil, where reproductive markets exhibit similar tensions and raise analogous questions about the social, ethical, and economic consequences of technological advances in assisted reproduction.

At the same time, formal accessibility appears as a necessary but not sufficient condition. While, as noted, there are regulatory frameworks and limitations, everyday experience reveals persistent forms of resistance. Tubal ligation, for example - although legally recognized in Argentina - remains subject to arbitrary restrictions that limit the actual access of users within the health system, as analyzed in the article by Galende Villavicencio included in this dossier.^31^

The legalization of abortion undoubtedly represents a milestone that has reshaped both public policies and cultures of care, though in highly uneven ways across countries. In Spain, voluntary termination of pregnancy has been legal since 2010, consolidating a broad regulatory framework and generating significant changes in health services and in the social perception of the right to choose. In Argentina, legalization in 2020 marked a historic step forward, reinforcing reproductive rights within a context of strong feminist activism. In contrast, Brazil maintains a highly restrictive framework: the 1940 Penal Code allows abortion only in two specific situations - when no other means exist to save the woman’s life, and in cases of sexual violence - reflecting a political and cultural context where criminalization continues to profoundly limit women’s reproductive autonomy and access. In 2012, a decision by the Federal Supreme Court extended this to include cases of anencephaly. Despite the illegality of abortion in Brazil, research shows that the practice is widespread. A national survey conducted in urban areas in 2010 and repeated in 2016 revealed that nearly one in five Brazilian women aged 40 had had at least one abortion.^32^ The illegality of abortion does not prevent it from occurring but rather deepens social inequalities: women in better socioeconomic conditions pay for abortions in private clinics, while most rely on misoprostol and, once bleeding begins, turn to public hospitals to complete the uterine evacuation - generally through curettage - and treat possible complications.^20^

A case currently before the Federal Supreme Court seeks to decriminalize voluntary abortion up to 12 weeks of gestation.^33^ A public hearing was held in 2018, and in 2023 only one favorable vote - that of the justice who introduced the case - was cast. This initiative faces strong resistance in the National Congress, whose majority is conservative, as well as from the most powerful actor, the Catholic Church, which is closely associated with conservative religious sectors, particularly evangelical and Kardecist spiritist groups. Public debate on abortion remains incipient.

In Argentina, however, the effective implementation of the law still reveals an uneven landscape marked by institutional resistance, regional disparities, and tensions between normative discourses and concrete practices. In this regard, the contribution of Briana Keefe-Oates and her team^34^ is particularly relevant: their work in developing questionnaires to evaluate public policies produces situated, feminist-informed data that make visible how rights are - or are not - materialized in specific local contexts. Similar difficulties can be observed in other contexts where, despite the existence of a legal framework, the voluntary interruption of pregnancy continues to be stigmatized.^35^

Moreover, the introduction of pharmacological technologies such as mifepristone - approved by Argentina’s National Administration of Drugs, Food and Medical Technology (ANMAT) and recommended by the WHO - illustrates how technical innovation shapes the expansion of rights and the configuration of collective health. The drug is not only a therapeutic tool but also a political actor that transforms access, redefines care pathways, and reconfigures the horizons of reproductive experience.

The various processes discussed here - from tubal ligation and abortion to surrogacy and the incorporation of pharmacological technologies - show that reproductive health takes shape within a field of human and non-human assemblages, traversed by legal, institutional, and cultural disputes. Far from constituting a homogeneous field, what emerges is a web of practices, regulations, and experiences that are assembled in situated ways, according to repronational histories and transnational dialogues.

## Intersections

Beyond analyzing the technological and health dimensions separately, this dossier seeks to explore their intersections: those spaces where human and non-human actors come together in networks of cooperation, conflict, and negotiation, producing unprecedented configurations of experience and meaning. The articles included in this dossier address different aspects and dimensions of these intersections.

Medication abortion constitutes a particularly significant example. Prior to its legalization, the circulation of misoprostol and mifepristone within feminist and professional networks enabled the development of safer and less stigmatized practices. As Lenta and Longo^36^ show, the medication acquired agency of its own: it became a producer of possible abortions, shaping subjectivities and collective trajectories that challenged stigma and enabled new forms of care. Here, technology appears not merely as an instrument but as a device capable of acting within a broader political and cultural assemblage.

Surrogacy offers another revealing case of intersection. The article by Ariza and Lima^37^ on the relationships between cisheterosexual married women seeking motherhood through Ukrainian surrogates shows how this process goes beyond the contractual framework to become a distributed and collaborative network. Drawing on Anika König’s notion of “reproductive entanglements,” inspired by Rayna Rapp, the authors show how actors, objects, and technologies converge as devices of connection, producing social and affective relationships that cannot be reduced to purely biomedical or legal dimensions.

In both cases, it becomes clear that reproductive technologies do not simply mediate between subjects and ends but act as constitutive elements of new realities. They are, in themselves, producers of subjectivities, kinship relations, and collective meanings.

The journey through these three dimensions - technologies, health, and intersections - makes it clear that reproduction is never a natural, given, or universal fact. It is, rather, the result of situated assemblages, traversed by regimes of knowledge and power, state regulations and market dynamics, cultural discourses and practices of resistance. Thinking about reproductive and reproductive health technologies from an intersectional perspective invites us to recognize that what is at stake is not only a set of biomedical procedures but the very definition of bodies, relationships, and possible futures.

Finally, as guest editors of this special issue, we would like to thank the authors, the editorial committee of *Salud Colectiva,* and the external reviewers who evaluated the articles included in this dossier. This collective effort has made it possible to bring together works that, we hope, contribute to understanding reproduction as a complex assemblage of actors, objects, discourses, and emotions, beyond its reduction to a merely biological or natural fact. Through this lens, we seek to offer tools to critically analyze and confront the challenges that reproductive health poses to our societies today.
